# Chrysin: Perspectives on Contemporary Status and Future Possibilities as Pro-Health Agent

**DOI:** 10.3390/nu13062038

**Published:** 2021-06-14

**Authors:** Monika Stompor-Gorący, Agata Bajek-Bil, Maciej Machaczka

**Affiliations:** 1 Department of Human Pathophysiology, Institute of Medical Sciences, University of Rzeszów, Warzywna 1a, 35-310 Rzeszów, Poland; maciej.machaczka@ki.se; 2 Faculty of Chemistry, Rzeszow University of Technology, 35-959 Rzeszów, Poland; abajek@prz.edu.pl; 3 Department of Clinical Science and Education, Division of Internal Medicine, Södersjukhuset, Karolinska Institutet, 118 83 Stockholm, Sweden

**Keywords:** chrysin, anticancer activity, neuroprotection, antioxidants, immunomodulators

## Abstract

Chrysin belongs to the group of natural polyphenols. It can be found, among others, in honey, propolis and fruits and has a wide range of biological activities, including the prevention of oxidative stress, inflammation, neurodegeneration and carcinogenesis. Being a part of the human diet, chrysin is considered to be a promising compound to be used in the prevention of many diseases, including cancers, diabetes and neurodegenerative diseases such as Alzheimer’s or Parkinson’s. Nevertheless, due to the low solubility of chrysin in water and under physiological conditions, its bioavailability is low. For this reason, attempts at its functionalization have been undertaken, aiming to increase its absorption and thus augment its in vivo therapeutic efficacy. The aim of this review is to summarize the most recent research on chrysin, including its sources, metabolism, pro-health effects and the effects of its functionalization on biological activity and pharmacological efficacy, evaluated both in vitro and in vivo.

## 1. Introduction

**Figure 1:**
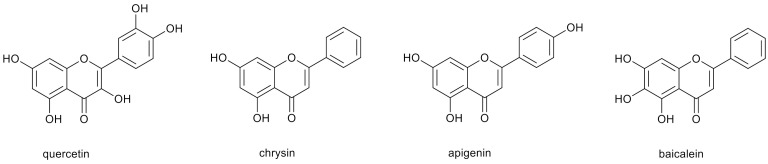
Chemical structures of selected polyphenols.

Polyphenolic compounds, including hydroxyflavones, are common components of many plants, fruits, medicinal herbs, flowers and dietary supplements [1,2]. Due to the presence of free hydroxyl groups in various configurations, they have high antioxidant activity [3]. To polyphenols belong such compounds as quercetin, chrysin, apigenin, baicalein and their derivatives (Figure 1). Their hydroxyl groups serve as reactive centers used for the functionalization of the compounds, aiming to increase their water solubility, bioavailability and biological activity [4,5].

Flavonoids with hydroxyl groups are important ingredients of humans’ daily diet; therefore, they are most thoroughly studied with respect to their influence on the human organism [6,7]. These studies have been preceded by several in vitro tests in which cell lines were used [8]. Natural hydroxyflavones have a range of biological activities, including anticancer [9], antioxidative [10], anti-inflammatory [11] antiallergic [12], hepatoprotective [13] and neuroprotective ones [14]. They prevent diabetes mellitus complications, such as retinopathy, which may lead to blindness [15]. Additionally, they have antimicrobial [16] and antiviral [17,18] properties. Because of their significant biological activities, they serve as basic substances for the development of new dietary supplements or foodstuffs intended for particular nutritional uses. Due to their pro-health properties, plant extracts rich in hydroxyflavonoids are used for the development of innovative recipes for desserts enriched with natural therapeutic substances, e.g., ice cream [19].

Taking into account the dynamic development of research on the role of polyphenolic compounds as therapeutic agents, the objective of this review is to summarize the current knowledge about natural sources of chrysin, its pharmacological activity and new ways of delivery. Moreover, recent reports about how chrysin can counteract the side-effects of some drugs used in conventional pharmacotherapy are summarized.

## 2. Sources of Chrysin

Chrysin (5,7-dihydroxyflavone) belongs to natural polyphenols, which are found among others in honey [20], propolis [21] and various medicinal plants and fruits (Table 1), such as bitter melon (*Momordica charantia*) [22] or the wild Himalayan pear (*Pyrus pashia*) [23]. In terms of chemical structure, chrysin belongs to the dihydroxyflavones, with the hydroxyl groups attached to the A aromatic ring (5-OH and 7-OH positions), the chemical formula C_15_H_10_O_4_ and molar mass, 254.241 g·mol^−1^. In plants, the precursor of chrysin is the amino acid phenylalanine, which is in the first step, converted to cinnamic acid by the action of the enzyme phenylalanine ammonia-lyase on the phenylpropanoid pathway.

The most recent research also confirms the presence of chrysin in *Diaphragma juglandis* fructus, walnut pellicle, the flowers of *Juglans regia* (common walnut) [24], the leaves and fruits of doum palms (*Hyphaene thebaica*) [25] and also the peel of passion fruit (*Passiflora edulis* Sims), where it is found in the form of glycoside (chrysin-8-C-(2″-*O*-β-6-deoxy-glucopyranosyl)-β-D-glucopyranoside, 0.35 mg kg^−1^) [26]. Chrysin was also identified in the medicinal herb Banxia Xiexin used in traditional Chinese medicine for the treatment of gastrointestinal diseases, where it is present in the form of 6-C-arabinoside-8-C-glucoside or as a glucuronic acid ester, i.e., chrysin-7-*O*-glucuronide [27]. In addition, chrysin glucuronides were also found in the aerial part of *Scutellaria schachristanica* [28]. Another source of chrysin is an endophytic fungus *Chaetomium globosum*, associated with a green marine alga (Chaetomorpha media) originating from India [29]. Chrysin and chrysin-7-*O*-β-D-glucopyranoside are the main ingredients of the Algerian plant *Cytisus villosus* Pourr, which has proven antiprotozoal activity against *Trypanosoma brucei* [30]. Chrysin in the form of 8-C-glucopyranoside is also an ingredient of *Salvadora persica*, the plant with antibacterial properties recommended by the World Health Organization for the production of the toothbrush fiber [31].

**Table 1:** The content of chrysin in the selected sources.

Product	Content of Chrysin	Ref.
Manuka honey	0.131 mg/100 g	[20]
Propolis extract	Acetone: 14.62 mg g^−1^ 70% EtOH: 18.64 mg g^−1^ 96% EtOH: 11.41 mg g^−1^	[21]
*Diaphragma juglandis* fructus	Up to 40 µg g^−1^	[24]
*Hyphaene thebaica*	0.083 mg g^−1^	[25]
*Chaetomium globosum*	13%	[29]
*Cytisus villosus*	4 mg kg^−1^	[30]

Because of the therapeutic properties of chrysin, research has been undertaken on its production in the hairy roots of plants, for example, *Scutellaria bornmuelleri*, obtained by genetic modification with the help of *Agrobacterium rhizogenes* [32]. The nutraceutical properties of plants rich in antiradical metabolites, including chrysin, encourage researchers to seek other methods of synthesis of such compounds, for example, using callus cell cultures obtained from different parts of the plants [33].

## 3. Metabolism of Chrysin

**Figure 2:**
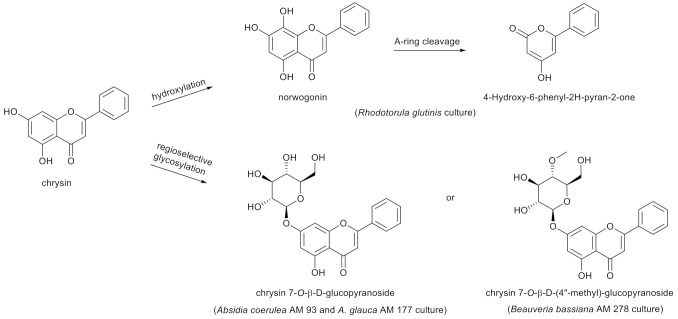
Metabolism of chrysin in *Rhodotorula glutinis*, *Absidia coerulea* AM93, *Absidia glauca* AM 177 and *Beauveria bassiana* AM 278 cultures.

An in vivo study in humans after oral administration of chrysin (400 mg) indicated that this is a compound with low bioavailability, depending mainly on fast metabolism in the gastrointestinal tract [34]. In search of the metabolic pathways of natural compounds, microbial and enzymatic transformations were employed. Receiving flavonoid derivatives of pharmaceutical importance by means of biotransformation is a commonly known method. Modifications of the main skeleton of flavonoids have strong influences on their biological activity. In some cases, biological methods of synthesis are the only possible way to achieve the compounds, which are very difficult to synthesize by chemical methods. The team of Liu et al. [35] identified a range of enzymes belonging to flavone hydrolases and methyltransferases, which are responsible for the synthesis of chrysin and its derivatives, such as baicalin and wogonin, in the culture of *Saccharomyces cerevisiae* yeasts. Another study [36] described that metabolism of chrysin in *Rhodotorula glutinis* over 72 h started from an initial C-8 hydroxylation of the substrate to norwogonin (31% yield), followed by the A-ring cleavage to 4-hydroxy-6-phenyl-2H-pyran-2-one as the final product. Meanwhile, ten-day biotransformations of chrysin in the cultures of *Beauveria bassiana* AM 278, *Aquilegia coerulea* AM 93 and *Absidia glauca* AM 177 led to chrysin 7-*O*-β-D-(4”-*O*-methyl)-glucopyranoside and chrysin 7-*O*-β-D-glucopyranoside in 14–23% yields (Figure 2) [37].

**Figure 3:**
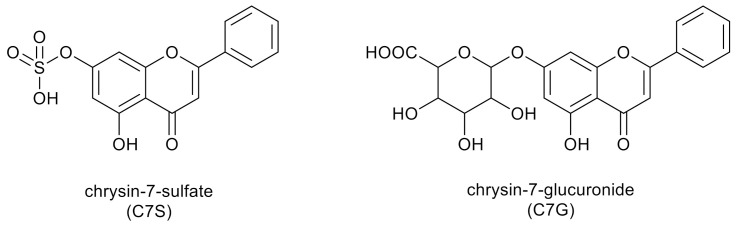
Two dominant products of chrysin metabolism in humans.

The two dominant products of chrysin metabolism in humans and in mice are chrysin-7-sulfate (C7S) and chrysin-7-glucuronide (C7G) (Figure 3) [34,38]. After the oral administration of chrysin (20 mg kg^−1^) to mice, the C_max_ of chrysin was only 10 nmol L^−1^, while 160 and 130 nmol L^−1^ peak plasma concentrations of C7S and C7G were quantified, respectively [38]. In another study, a 400 mg dose of chrysin was administered orally to healthy human volunteers, after which C7S reached approximately 30-fold higher AUC0–∞ values compared to chrysin (420–4220 ng·mL^−1^·h vs. 3–16 ng·mL^−1^·h, respectively) [34]. Based on previous studies, chrysin is a potent inhibitor of some biotransformation enzymes (e.g., CYP3A4, CYP2C9 and xanthine oxidase) and is also able to affect drug transporters (e.g., P-glycoprotein) [39]. Additionally, Bojić et al. [40] proved the inhibitory activity of chrysin to cytochrome P_450_ monooxygenase CYP1A2.

In an ex vivo study by Labib et al. [41], it was demonstrated that chrysin is not metabolized by the pig intestinal microflora, unlike its structural analogs naringenin, quercetin or hesperetin, which undergo degradation to low-molecular-weight compounds (3,4 dihydroxytoluene, phloroglucinol). In the case of hesperetin, it is preceded by the *O*-demethylation to eriodictyol.

## 4. Biological Activity

### 4.1. Anticancer Activity of Chrysin and Its Derivatives

Among the pharmacological activities of chrysin, its anticancer properties are well documented [42]. It acts through the induction of apoptosis and inhibition of cancer cell migration [43,44]. Its antitumor properties were proven for various malignancies, including prostate cancer (DU 145, PC-3) [45], breast cancer [46], lung cancer (A549), liver cancer (HepG2), colon cancer (SW480) [47] and pancreatic cancer (SW1736, 8505C) [48], and also epidermoid carcinoma (A431) [49], glioblastoma (T98, U251, U87) [50] and human uveal melanoma (SP6.5, M17) [51]. According to the Salama and Allam study [52], chrysin and daidzein exerted anticancer activity against SW620 cells, which was associated with a decrease in the protein expression of p-ERK/ERK and p-AKT/AKT.

Moreover, the anticancer activity of the amino acid chrysin derivatives obtained by chemical synthesis has been proven [53]. Thus, the development of new chrysin derivatives with potentially better antitumor properties is reasonable [54,55,56].

Some long-chain ester derivatives of chrysin are also known to have good biological activity. An example is 7-*O*-myristyl chrysin, a compound with a flexible structure, considerably good solubility and activity against liver cancer cell lines 5.4 times greater than chrysin (IC50 - compound concentration leading to 50% inhibition of cell proliferation was 74.97 μmol L^−1^) [57].

Meanwhile, the replacement of the oxygen at C-4 in chrysin with selenium resulted in increased antioxidant activity, both in aqueous and lipid media. It was also confirmed that the higher pH, the faster the reaction of the selenium chrysin derivatives with the HOO˙ radical [58].

**Figure 4:**
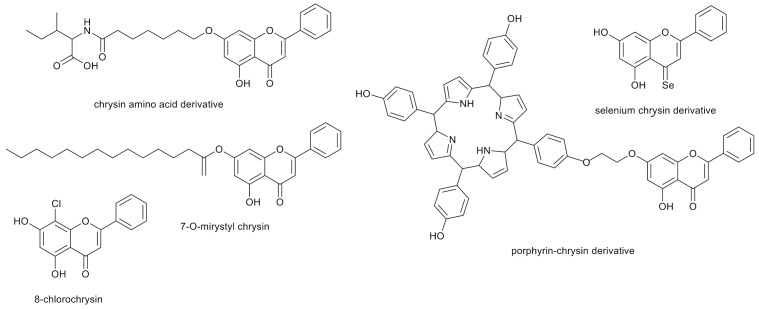
Examples of chrysin derivatives with proven anticancer activity.

Another method of modification is halogenation. In the most recent research, it was shown that substitution of chrysin with chlorine increases the binding affinity to human protein kinase hCK2α, which is a therapeutic target for new inhibitors used in the treatment of cancer due to the strong correlation between malignancy and abnormally high activity of this protein in cancer cells. It was observed that 8-chlorochrysin (Figure 4) had stronger binding activity to hCK2α than the reference CK2 inhibitor, 4,5,6,7-tetrabromo-1H-benzotriazole [59].

Moreover, chrysin and its porphyrin derivatives can be used in the non-invasive photodynamic therapy of human gastric cancer cells (MGC-803) and human cervical cancer cells (HeLa) [60].

### 4.2. Influence of Chrysin on Side Effects Associated with Pharmacotherapy

Nowadays, the goal of medical science is to find and identify safe compounds, free from side-effects, which may be used as therapeutics in humans. It was proven that natural substances may be effective adjuvants in the treatment of many diseases, not only in the case of immunodeficiency but also in the prevention of a range of disorders in patients with properly functioning immune systems.

Due to the strong interaction of chrysin metabolites (mainly chrysin-7-sulfate) with human serum albumins (HAS), the intake of chrysin with food may affect the albumin-binding properties of some drugs [61]. In this way, it affects the pharmacokinetics, biological activity and half-life of a drug, as it is the amount of a free drug in the blood (unbound to plasma proteins) that has a therapeutic effect.

Commonly used anticancer drugs, such as mitomycin C, by interaction with biological molecules, can cause genetic damage in healthy cells, for example, in the liver, kidneys or bone marrow. In addition, they can increase the activity of intracellular antioxidant enzymes and increase lipid peroxidation. Meanwhile, administration of chrysin in a dose of 40 mg kg^−1^, 24 h prior to the treatment with mitomycin C, caused regression of the genotoxic effect, which also resulted partly from the high antioxidant activity of chrysin [62].

Currently, there is ongoing research on using chrysin in combination therapy, to enhance the efficiency of chemotherapeutics such as docetaxel [63], cisplatin and camptothecin [64]. Moreover, chrysin regulates abnormal changes in enzyme activities induced by commonly used anticancer drugs, such as cyclophosphamide. This is confirmed by the results of the study on the effect of chrysin on the regulation of the pentose phosphate pathway enzymes (playing a pivotal role in cancer cell proliferation) and the enzymes of the reduced glutathione and thiorodexin system, which take part in intramolecular ROS removal [65].

Similar conclusions were drawn by the group of Taslimi et al. [66]. Chrysin administered to rats (25 and 50 mg kg^−1^) seven days prior to treatment with a single dose of cyclophosphamide (200 mg kg^−1^) considerably reduced the toxic effects of this drug by regulating the activity of metabolic enzymes of the liver (e.g., carbonic anhydrase, aldose reductase, paraoxonase-1,α-glycosidase, acetylcholinesterase), heart and brain (butyrylcholinesterase). Administered in combination with methotrexate, chrysin has a protective effect on methotrexate-induced testicular damage in rats [67]. Darendelioglu et al. [68] proved that chrysin reversed the harmful side-effects of some nonsteroidal anti-inflammatory drugs, such as diclofenac. The in vitro study was conducted on the SH-SY5Y human neuroblastoma cell line. Moreover, chrysin, probably due to its antioxidant activity, ameliorates isoniazid-induced changes, such as brain oxidative damage, inflammation and apoptosis [69]. According to an in vivo study on rats, chrysin (in a dose of 50 µg mL^−1^) co-administered with paracetamol increased the absorption of this drug [70].

### 4.3. Hepatoprotective Effects of Chrysin

The most important risk factors for liver damage include excessive alcohol consumption, pharmaceuticals and hepatoxic substances, and also an unhealthy diet and being overweight or obese. In clinical practice, for evaluation of the stage of liver pathology, the most common biochemical tests are used. They include the activity of serum alanine aminotransferase (ALT), aspartate aminotransferase (AST) and alkaline phosphatase (ALP), the concentrations of bilirubin and albumins in plasma and the international normalized ratio (INR).

According to the most recent reports, chrysin has a positive effect on the liver by protecting the cells from toxic substances. It induces the secretion of very-low-density lipoproteins (VLDL) and reduces liver fat accumulation in non-alcoholic fatty liver disease (NAFLD) caused by a diet deficient in methionine and choline [71]. It also prevents hepatic necrosis. The group of Mohammadi et al. [72] proved that chrysin decreased the levels of liver enzymes (ALT, AST and ALP), which were previously increased by the use of acetaminophen. It has a protective effect on HepG2 hepatic cells damaged by ethanol. Chrysin, along with other ingredients of propolis, suppresses specific signaling pathways, i.e., ERK1/2 phosphorylation, AHR nuclear translocation and CYP1A1 expression [73]. Administration of chrysin prevents oxidative damage in the liver and kidneys induced by long-term alcohol consumption. Tests on Wistar rats proved that chrysin restored normal levels of oxidative stress markers, such as glutathione peroxidase, catalase and glutathione reductase [74]. Due to the proven antioxidant, anti-inflammatory and antifibrotic properties of chrysin, it is a promising agent for the treatment of non-alcoholic fatty liver disease (NAFLD) [75]. On that basis, Fatemi et al. [76], in an in vivo study, determined the mechanisms associated with the possible protective effects of chrysin against sodium arsenite-induced liver damage.

### 4.4. Chrysin in Skin Diseases

Chrysin has the ability to attenuate psoriasis-like skin lesions [77]. Its hydroxyethylated derivatives obtained by gamma irradiation may find an application in the treatment of atopic dermatitis because they decrease the levels of pro-inflammatory cytokines IFN-γ, IL-5, IL-4 and IL-17 [78]. In addition, by targeting IκB kinase in the atopic dermatitis-like inflammatory microenvironment, chrysin inhibits NF-κB-dependent CCL5 transcription [79].

Administration of chrysin to BALB/c mice with dermatitis induced by 2,4 dinitrochlorobenzene and the house dust mite resulted in reduced levels of blood histamine and inhibited the secretion of Th1, Th2, Th17, CCl17 and CCl22 cytokines. In addition, chrysin inhibited the expression of IL-33 [80], and therefore, may find an application in the treatment of atopic dermatitis and skin allergy diseases. Similar conclusions were drawn by Song et al. [78], who proved the anti-inflammatory influence of the chrysin derivatives, obtained by gamma irradiation of chrysin on atopic dermatitis-like skin lesions in Balb/c mice. Moreover, it has been confirmed that chrysin protects human epidermal keratinocytes from UVA- and UVB-induced damage [81]. What is more, chrysin also protects the skin from photoaging and melanogenesis [82].

### 4.5. Chrysin in Neurodegenerative and Eye Diseases

Polyphenolic compounds have neuroprotective properties, improving processes of memorization. Chrysin also has such an activity and improves the processes involved in memory [83]. It regulates neurogenesis in memory loss due to aging [84]. It has also neuroprotective effect on SH-SY5Y neuronal cells treated with diclofenac [68]. The neuroprotective activity of chrysin may be used in the future to treat neurodegenerative diseases, including Parkinson’s disease [85].

The accumulation of D-galactose induces brain aging due to oxidative stress and inflammation, leading to neuronal cell damage and memory loss. The administration of chrysin at the doses of 10 or 30 mg kg^−1^ in rats with D-galactose-induced memory disorders reversed neuronal pathological changes and attenuated the memory impairments associated with aging [84].

The most recent research suggests that chrysin may be used to treat eye diseases leading to blindness, such as macular degeneration or choroidal neovascularization [86], and also autoimmune diseases [87].

Because of the proven in vivo activity of chrysin in the inhibition of choroidal neovascularization (CNV) and in downregulation of HIF-1α and VEGF expression in rats with diode laser-induced CNV, chrysin is a promising agent to combat age-related macular degeneration (AMD) that may lead to vision loss in elderly people [86].

### 4.6. Other Biological Effects of Chrysin

Chrysin has an influence on the biosynthesis of sphingolipids, which may be associated with a reduction of oxidative stress that depends on levels of ceramides in the organism [88]. It has antiviral properties against such viruses as human rhinoviruses (HRV) [89], influenza H1N1 [90], coxsackievirus B3 (CVB3) [91] and others. It also has an antiallergic activity, which was confirmed in a study on its influence on airway inflammation. Administered at a dose of 50 mg kg^−1^ daily in a mouse model, chrysin significantly suppressed airway hyperresponsiveness to acetylcholine chloride, caused by ovalbumin. In addition, chrysin decreased total immunoglobulin E (IgE) levels in serum and the total number of inflammatory cells and eosinophils in BALF [92]. Because of its antiproliferative and pro-apoptotic activity, chrysin plays a protective role in benign prostatic hyperplasia associated with testosterone, commonly affecting men in their sixties [93]. According to the newest reports, reduction of oxidative stress achieved after administration of chrysin leads to the conclusion that this compound can be employed in the treatment of acute pancreatitis [94]. Chrysin also has antinociceptive properties in diabetes mellitus complications, considerably affecting quality and length of life, such as diabetic neuropathy [95].

What is more, chrysin has the ability to regulate metabolism. It shows a moderate ability to block the transcripts that regulate the rate-limiting enzymes involved in the biosynthesis and absorption of cholesterol [96]. According to the most recent reports, chrysin can reduce lipid accumulation by downregulating the inflammation-related target proteins ANXA2 and HSP-60, and thus may play a significant role in the treatment of obesity [97]. It also has proven anti-anxiety activity; however, in the case of chronic use, it may be addictive, similar to benzodiazepines [98]. Rodríguez-Landa et al. [99], in an in vivo study, demonstrated that chrysin (2 mg kg^−1^) prevents anxiety-like behavior by the action on γ aminobutyric acid-A (GABA_A_) receptors. Differences in the mechanisms involved in GABA receptors’ modulation by some flavonoids compared with classic benzodiazepine modulation were reported by Goutman et al. [100]. According to the results of this study, chrysin, in the micromolar range, inhibits ionic currents mediated by GABA_A_ and GABA_C_ receptors.

Chrysin may find an application in the treatment of gynecological diseases, such as endometriosis, which may lead to infertility. Research with the use of human endometriotic cells derived from the cervix (End1/E6E7) and vagina (VK2/E6E7) revealed that chrysin suppressed the proliferation and induced apoptosis of these cells by affecting the cell cycle, changing the cytosolic calcium level, affecting the generation of reactive oxygen species and by inactivating the PI3K signaling pathways [45].

Kseibati et al. [101] described a positive effect of chrysin administered orally at a dose of 50 mg kg^−1^ on bleomycin-induced pulmonary fibrosis. Chrysin reduced hydroxyproline content, decreased expression of transforming growth factor-β1 (TGF-β1) protein, reduced the activity of lactate dehydrogenase (LDH) and decreased lipid peroxidation.

Multiple sclerosis is a chronic disease of the central nervous system, which may cause serious disability. The exact cause of multiple sclerosis is unknown, though it is known that short, non-coding, single-stranded microRNA molecules are important expression regulators of the genes associated with MS-pathophysiology. Del Fabbro et al. [102] demonstrated that 25-day chrysin supplementation (20 mg kg^−1^) decreased microRNAs-21 and 155 expression levels changed in experimental autoimmune encephalomyelitis (EAE), induced by myelin oligodendrocyte glycoprotein (35–55) peptide in C57BL/6 mice.

Plant extracts containing chrysin have valuable antimicrobial properties [103], which served as inspiration for the development of innovative packaging materials for industrial purposes containing substances with antioxidant and antibacterial properties, such as chrysin, apigenin and lutein. Although these materials have reduced tensile strength, they have enhanced UV–vis light barrier and antibacterial activity. The film containing chrysin had the highest antibacterial activity against Escherichia coli, Salmonella typhimurium, Staphylococcus aureus and Listeria monocytogenes [104]. It is common knowledge that propolis and various kinds of honey containing chrysin have an antifungal activity [105,106,107], which depends on the thermal processing of honey.

#### Pharmacological Effects of Chrysin Complexes

One of the techniques used to improve the solubility of poorly soluble substances is complexation, including the formation of chrysin complexes with metal ions.

The complexation of chrysin with transition metal ions leads to the generation of novel metallodrugs with improved pharmacological and biochemical properties. Recently, it was described that, for example, chrysin complexes with zinc (II) have an antioxidant potential [108], with magnesium (II) have antitumor activity against lung cancer (A549) and are non-toxic against normal human fibroblasts [109], and with La (III) are characterized better anticancer and DNA binding effects than chrysin [110]. According to the physiological studies described by Ravishankar et al. [111], ruthenium-conjugated chrysin analogs may be a basis to develop new antithrombotic drugs (Table 2). Furthermore, Ravishankar et al. [111] and Marques et al. [112] described novel ruthenium conjugates of chrysin as anticancer composites.

The Al(III), Ga(III) and In(III) complexes with chrysin and also the titanium(IV), iron(III) and manganese(II) complexes of chrysin-4′-sulfonate are known, but their activity has not been tested [114,115]. Furthermore, the complexation of chrysin with Pb(II) [115] and vanadyl(IV) [116] was described with antioxidant activity.

Another method is complexation between chrysin and amino-appended β-cyclodextrins. This is proven to increase the solubility to 4411.98 µg mL^−1^. The complexation also improved the antioxidant activity and cytotoxicity against cancer cell lines (A549, HT-29, HCT116) [122]. Ignat et al. [122] described biocompatible chrysin-β-cyclodextrin complexes characterized improved pharmacological effects.

## 5. Innovative Ways of Chrysin Delivery

Because chrysin in the form of aglycone is poorly soluble in water [123], the in vivo studies on improvement of its absorption by using various carriers have been undertaken. It was confirmed that chrysin complexes with methylated β-cyclodextrin, obtained by lyophilization, were highly effective to enhance chrysin permeability through the Caco-2 monolayers [124], and were not cytotoxic to the cancer cells (Table 3). Also, the cyclodextrin complexes of chrysin containing β-cyclodextrin (β-CD), hydroxypropyl-β-cylcodextrin (HPBCD) and sulfobutylether-β-cylcodextrin (SBECD) after 30 min of incubation were not toxic to Caco-2 cells at the concentration of up to 100 µM. Whereas, at the concentration of 200 µM the cell viability decreased below 80%. There are also attempts to synthesize chrysin-polyvinylpyrrolidone microparticles by encapsulation using the supercritical antisolvent (SAS) process, in order to increase therapeutic efficacy in cancer treatment. The most recent research indicate that such constructs have higher antiproliferative activity against MDA-MB-231 breast cancer cell line compared with chrysin, which is attributed to their better solubility under physiological conditions [125]. Chrysin nanocapsules based on polylactic-glycolic acid (PLGA) can find application also as effective anti-glycemic and anti-hyperlipidemic agents [126]. There is also ongoing research into employment of phospholipid chrysin carriers for control of blood sugar in mice with type 2 diabetes [127]. Moreover, chrysin in combination with well-known anticancer chemotherapeutics used in the first-line cancer treatment, such as 5 fluorouracil, co encapsulated with the use of PLGA-PEG-PLGA copolymer may be an important strategy to improve therapeutic efficacy in cancer therapy, including human colon cancer (HT-29) [128]. There is also some evidence that chrysin-loaded nanoliposomes, administered at the doses of 2.5 and 5 mg kg^−1^ to mice (*in vivo* study) alleviate the symptoms of the cadmium-induced toxicity. They improve deposition of antioxidant minerals, modulate liver enzymes, alleviate hepatic oxidative stress, and improve the morphohistological structure of jejunum (height and width of the intestinal villi) [129].

The nano-encapsulation of curcumin and chrysin enhanced delivery of these compounds to SW480 colorectal cancer cells [130]. Furthermore, it is known activity of chrysin-PLGA nanoparticles, orally administrated, in ameliorating allergic asthma progression [131].

Also co-encapsulated curcumin and chrysin in PLGA-PEG nanoparticles inhibited the melanoma B16F10 tumor growth and decreased gene expression (TERT – ang. *telomerase reverse transcriptase*, MMP-9 - ang. *matrix metallopeptidase 9,* and MMP-2) [132]. Halevas et al. [133] prepared novel chrysin-loaded poly(ε-caprolactone) and poly(3-hydroxybutyrate) microcarriers, containing the poly(vinyl alcohol) as stabilizer with sizes between 2.4 and 24.7 µm.

Designing new delivery systems for chrysin is improving its pharmacological properties and biodistribution. Despite the many advantages of the carriers used heretofore, they also have numerous disadvantages and limitations. One of them is quick elimination from the circulation or unfavorable pharmacokinetic features. Protein carriers, in turn, are characterized by a short biological half-life, low stability under physiological conditions and immunogenicity. Therefore, it becomes necessary to undertake detailed research on the use of, for example, non-toxic cosolubilizers as auxiliary substances, as well as tropic molecules that recognize the target site, in order to support the future development of personalized medicine.

## 6. Conclusions

Polyphenols are a very large group of natural compounds that are a part of the human diet. Due to their structural diversity, they also have diverse pharmacological activities. Chrysin, which is found among others in citrus fruits, honey and propolis, has a wide range of biological activities, including anticancer, antioxidant, hepatoprotective, antiviral, neuroprotective and anti-anxiety ones. It is also an adjuvant for some drugs.

Several investigations have been aimed at enhancing chrysin’s water solubility to achieve higher plasmatic concentrations and decrease the gastric side-effects of some pharmaceuticals. New chrysin delivery formulations have shown successful results.

As methods for improvement, the synthesis of effective chrysin analogs or preparations dedicated to different application routes—such as cancer, dermal or oral route formulations—have also been proposed.

These formulations include different types of polymers and other substances intended to deliver chrysin slowly to the target site. In this area, the use of nanotechnology becomes of special relevance, and several attempts have been undertaken to improve the chrysin release profile.

Although the metabolic pathways of chrysin—both in vitro and in vivo—are known, little is known about the influence of its biotransformation products on the human body. A detailed biological study is needed for new chrysin derivatives with high antioxidant activity.

## Figures and Tables

**Table 2:** Novel chrysin complexes and their biological effects.

Complex	Activity	Ref.
Zinc(II)-chrysin	Antioxidant potential	[108]
Magnesium(II)-chrysin	Antitumor effect against lung cancer A549 cells	[109]
Ru-thio-chrysin complex	Four-fold greater inhibition of platelet function and thrombus formation in vitro than chrysin	[111]
Ruthenium(II) trithiacyclononane complexes of chrysin	Anticancer activity MG-63 (osteosarcoma), PC-3 (prostate) IC_50_ = 146.2 µM, MCF-7 and MDA-MB-231 (breast adenocarcinoma) IC_50_ = 180.6 µM	[112]
Ga(III)–chrysin–imidazole complex displayed the highest anticancer efficacy against all cancer cell lines with IC_50_ values in the low micromolar range (<1.18 μM), a result worth further investigation	IC_50_ values in the low micromolar range (<1.18 μM)	[113]
Al(III), Ga(III), In(III) complexes	Not tested	[114]
Titanium(IV), iron(III) and manganese(II) complexes of chrysin-4′-sulfonate	Not tested	[115]
Chrysin-Pb(II)	Antioxidant activity	[116]
Chrysin-VO(IV)	Antitumoral, antioxidant	[117]
Cu(II) complexes of chrysin with 2,2′–bipyridine and substituted 1,10–phenanthrolines	Antioxidant activity	[118]
Cu(II)-chrysin Cu(II)-chrysin-1,10-phenanthroline Cu_2_(L)_2_(phen)_2_](NO_3_)_2_·MeOH Cu(II)-chrysin-2,2′-bipyridine Cu(L)(bipy)(MeOH)](NO_3_)·MeOH	A549 and H1299 lung cancer cell lines after 24 h of exposure exhibit enhanced solubility and bioavailability and also improved cytotoxic and genotoxic activity compared to free chrysin	[119]
Oxidovanadium (IV) complexes with chrysin	Anticancer activity	[120]
Chrysin-amino-appended β-cyclodextrins	Anticancer (A549, HT-29, HCT116) and antioxidant activity	[121]
Chrysin complexes with two cyclodextrins (CDs)-(2-hydroxypropyl)-β-cyclodextrin (HPBCD) and random methyl-β-cyclodextrin (RAMEB)	Anti-inflammatory antioxidant anti-fibrotic effects	[122]

**Table 3:** New chrysin delivery systems.

Pharmaceutical Form	Physicochemical Characteristics	Activity	Ref.
Chrysin complexes with methylated β-cyclodextrin	Solubility increment: 4.37–8.04	High peremetion through the Caco-2 monolayer	[124]
chrysin-polyvinylpyrrolidone sub-microparticles	size of 273.7 nm	antiproliferative effect (MDA-MB-231 cells)	[125]
PLGA-chrysin nanocapsules	Size: 176 nm Polydispersity index: 0.22 negative zeta potential Drug entrapment efficiency: 87.1%	Anti-glycemic and anti-hyperlipidemic agent	[126]
Phospholipid-chrysin carriers	Egg phospholipid: chrysin 1:3	Antidiabetic	[127]
Chrysin–5-fluorouracil–PLGA-PEG-PLGA nanocapsules	Combination index: 0.35 Zeta potential (mv): −12.8 ± 4.0 Size: 40 nm	Anticancer (HT-29 cells)	[128]
Nanoliposome-loaded chrysin (NLC)	Size: 185.1 nm Polydispersity index: 0.26	Alleviated the symptoms of cadmium-induced toxicity in mice in doses 2.5 and 5 mg/kg	[129]
PLGA-PEG-chrysin nanoparticles	Size: 50–140 nm	Enhanced delivery to SW480 colorectal cancer cells IC_50_ = 42 µM (24 h) IC_50_ = 36 µM (48 h) IC_50_ = 33 µM (72 h)	[130]
Chrysin-loaded PLGA	Size: 65–90 nm (TEM) Size: 77 nm (AFM) Polydispersity index: 0.084 Zeta potential (mv): −9.33 ± 0.5 (pH = 6.8)	CHR-NPs squelched OVA-induced pulmonary histopathological alterations, inflammatory cell influx, Th2-cytokine IL(-4, -5 and -13) BALF levels and serum (IgE), as well as pro-inflammatory cytokines (TNF-α,IL-1β, IL-6, IL-18) in both serum and lung tissue more potently than free chrysin (50 mg/kg body weight)	[131]
Chrysin-loaded PLGA-PEG nanoparticles	Size: 233 nm Polydispersity: 0.145 Zeta potential (mv): −6.2 ± 2.5	Antitumor (B16F10 cells)	[132]
Chrysin-loaded poly(ε-caprolactone) and poly(3-hydroxybutyrate) microcarriers, containing the poly(vinyl alcohol)	Size ranging between: 2.4–24.7 µm Zeta potential (mv): (−18.1)–(−14.1)	Anticancer (MDA-MB 231 cells) IC_50_ = 149.19 µM IC_50_ = 312.18 µM Low (up to 2%) hemolytic percentages at concentrations between 5 and 500 µg·mL^−1^	[133]

IC_50_—compound concentration leading 50% inhibition of cell proliferation. CHR-NPs—chrysin nanoparticles. BALF—bronchoalveolar lavage fluid. TEM—transmission electron microscopy. AFM—atomic force microscope.

## Data Availability

All data are publicly available.

## Abbreviations

ALP	alkaline phosphatase
ALT	alanine aminotransferase
ANXA2	annexin A2
AST	aspartate aminotransferase
BALF	bronchoalveolar lavage fluid
β-CD	β-cyclodextrin
CCL5	C-C motif chemokine ligand 5
CNV	choroidal neovascularization
ERK	extracellular signal-regulated kinase
HAS	human serum albumin
HPBCD	hydroxypropyl-β-cylcodextrin
HRV	human rhinoviruses
INR	international normalized ratio
LDH	lactate dehydrogenase
NAFLD	non-alcoholic fatty liver disease
NF-ĸB	nuclear factor kappa B cells
PEG	polyethylene glycol
ROS	reactive oxygen species
TGF-β1	transforming growth factor beta 1
VEGF	vascular endothelial growth factor
VLDL	very-low-density lipoproteins
